# Retraction: Identification of a Novel Gammaretrovirus in Prostate Tumors of Patients Homozygous for R462Q *RNASEL* Variant

**DOI:** 10.1371/annotation/7e2efc01-2e9b-4e9b-aef0-87ab0e4e4732

**Published:** 2012-09-18

**Authors:** Anatoly Urisman, Ross J Molinaro, Nicole Fischer, Sarah J Plummer, Graham Casey, Eric A Klein, Krishnamurthy Malathi, Cristina Magi-Galluzzi, Raymond R Tubbs, Don Ganem, Robert H Silverman, Joseph L DeRisi

In light of the findings from a recent study “In-Depth Investigation of Archival and Prospectively Collected Samples Reveals No Evidence for XMRV Infection in Prostate Cancer” (http://dx.plos.org/10.1371/journal.pone.0044954) and others in the field, the editors of PLOS Pathogens have issued a retraction of this study. The association of XMRV with prostate cancer has now been thoroughly refuted. Although the original finding of a novel gammaretrovirus, XMRV, with the use of a pan-viral detection microarray is valid, and sequencing and phylogenetic characterization of the virus still stands, the editors agree that it is clear that XMRV found in this study is laboratory-derived and there is no association of XMRV with prostate cancer. As a result the paper was retracted from PLOS Pathogens on September 18th, 2012.

